# Computer model for the cardiovascular system: development of an e-learning tool for teaching of medical students

**DOI:** 10.1186/s12909-017-1058-1

**Published:** 2017-11-21

**Authors:** David Roy Warriner, Martin Bayley, Yubing Shi, Patricia Victoria Lawford, Andrew Narracott, John Fenner

**Affiliations:** 1 Mathematical Modelling in Medicine Group, Department of Infection Immunity and Cardiovascular Disease, University of Sheffield, The Medical School, Room OU140, O Floor, Beech Hill Road, Sheffield, S10 2RX UK; 2 Department of Cardiology, Northern General Hospital, Sheffield Teaching Hospitals, Herries Road, Sheffield, S5 7AU UK; 3 Department of Scientific Computing, Royal Hallamshire Hospital, Sheffield Teaching Hospitals, Glossop Road, Sheffield, S10 2JF UK

**Keywords:** Cardiology, Cardiovascular science, E-learning, Virtual patients

## Abstract

**Background:**

This study combined themes in cardiovascular modelling, clinical cardiology and e-learning to create an on-line environment that would assist undergraduate medical students in understanding key physiological and pathophysiological processes in the cardiovascular system.

**Methods:**

An interactive on-line environment was developed incorporating a lumped-parameter mathematical model of the human cardiovascular system. The model outputs were used to characterise the progression of key disease processes and allowed students to classify disease severity with the aim of improving their understanding of abnormal physiology in a clinical context. Access to the on-line environment was offered to students at all stages of undergraduate training as an adjunct to routine lectures and tutorials in cardiac pathophysiology. Student feedback was collected on this novel on-line material in the course of routine audits of teaching delivery.

**Results:**

Medical students, irrespective of their stage of undergraduate training, reported that they found the models and the environment interesting and a positive experience. After exposure to the environment, there was a statistically significant improvement in student performance on a series of 6 questions based on cardiovascular medicine, with a 33% and 22% increase in the number of questions answered correctly, *p* < 0.0001 and *p* < 0.001 respectively.

**Conclusions:**

Considerable improvement was found in students’ knowledge and understanding during assessment after exposure to the e-learning environment. Opportunities exist for development of similar environments in other fields of medicine, refinement of the existing environment and further engagement with student cohorts. This work combines some exciting and developing fields in medical education, but routine adoption of these types of tool will be possible only with the engagement of all stake-holders, from educationalists, clinicians, modellers to, most importantly, medical students.

**Electronic supplementary material:**

The online version of this article (10.1186/s12909-017-1058-1) contains supplementary material, which is available to authorized users.

## Background

Over the last decade medical education has moved away from a traditional didactic, system with lecture-based teaching to more contemporary self-directed learning supported by case-based discussions stimulated by problem-based curricula. More recently, electronic media, such as web-based online supporting material, live streaming of lectures and downloadable applications have emerged as valuable teaching tools [1–3]. Yet, despite making use of modern platforms, one problem remains the same: these are often passive ways of learning, lacking interactive or integrated assessment elements (of students learning). It is now acknowledged that to maximise medical students learning, in terms of effectiveness and retention, learning should be active e.g. problem based, preferably interactive (especially for deep learners) and clinically relevant and that it is an unavoidable truth that assessment drives learning [4–10]. The use of simulated (modelled) patients, in virtual learning environments, is becoming common-place in both undergraduate and post-graduate medical teaching [11]. Simulations enable commonly encountered and potentially serious conditions to be explored, in a safe and non-threatening environment, with instant feedback on performance. The use of simulated patients adds to the richness of a learning experience and places theory in a clinical context [12].

The author’s aim therefore was to exploit local expertise in the Medical School (MS) at the University of Sheffield (UoS) in cardiovascular modelling, clinical cardiology and e-learning, to create an environment, incorporating both interactive and assessment elements, to assist undergraduate medical students to develop a good understanding of key physiological and pathophysiological concepts relating to the cardiovascular system. The environment used easy to understand cardiovascular system (CVS) models, up-to-date clinical information and realistic, simulated, clinical cases, all based on the needs of the current medical curricula.

The science of the cardiovascular system and the art of practicing cardiology are knowledge and skills that medical students and junior doctors must master. Cardiac disease is common, being among the most frequent reasons for a patient to present at hospital, with symptoms as varied as chest pain, breathlessness, palpations or dizziness. The practice of cardiology is complex, and one of the most rapidly growing subspecialised disciplines in all of hospital medicine, with a huge associated panoply of imaging modalities, interventional techniques and electrical devices. Such complexity suggests the requirement for hi-fidelity learning modalities.

Whilst there are a wide range of on-line case-based teaching platforms offered by journals e.g. British Medical Journal (BMJ) and learned societies such as the European Society of Cardiology (ESC) (http://learn.escardio.org/Default.aspx), there are no tools such as those developed in dermatology by Wahlgren et al. (2006) that target undergraduates [13, 14]. The work by Wahlgren et al. (2006) developed a computerised interactive case simulation system, where the “student selects a patient and proposes questions for medical history, examines the skin, and suggests investigations, diagnosis, differential diagnoses and further management” based on authentic cases with images from real patients [13]. Whilst students reported this facilitated their learning, there was no actual assessment of their learning. More specifically to cardiovascular science, Dassen et al. [15], developed the “CircAdapt” mathematical model for first year medical students in Maastrict, which was a lumped parameter model (LPM) describing the hemodynamic interaction between the left and right ventricle, simulating the pulmonary and systemic circulations. This model enabled students to interact with model parameters, such as heart rate or peripheral resistance to mimic different physiological states, such as exercise or hypovolemic shock. This study did not report any assessment of learning, integration with actual clinical cases or provide student feedback on the model.

Many approaches exist to develop mathematical models of the circulation, governed by energy, mass and momentum conservation. Lumped parameter, or zero-dimensional, circulation models are often described in terms of hydraulic-electrical analogues; just as in an electrical circuit the voltage gradient determines the flow of current, the flow of blood in the cardiovascular system is determined by the pressure gradient and so a LPM can be used to simulate physiology and pathophysiology of the cardiovascular system, furthering understanding. The benefits of an LPM approach is that they typically provide simple, elegant and quick solutions and require relatively few model parameters which can typically be related directly to concepts familiar to the clinician.

Considering this context, an interactive model-based environment, which explains the fundamentals of cardiovascular physiology and pathophysiology, placed in a relevant clinical context for undergraduates, with built in assessment, could help the transition from passive student to responsible clinician.

### Aims

To incorporate a LPM into an e-learning environment (ELE) to act as a tool for understanding CVS physiology, map the progression of key disease processes and allow students to classify severity of disease, which will all enable a greater understanding of abnormal cardiovascular physiology, in a clinical context which is appealing to students.

## Methods

### Lumped parameter model

**Fig. 1:**
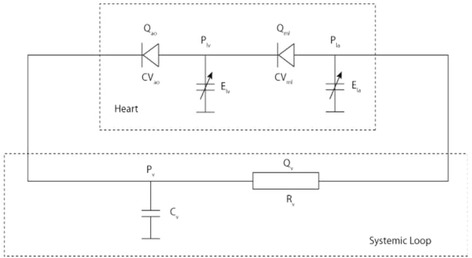
Lumped parameter model of the (left) heart, with left atrium (Ela), left ventricle (Elv), aortic valve (CVao) and mitral valve (CVmi) and systemic loop (circulation), with resistor (Rv) and capacitor (Cv). Note P and Q, denote pressure and flow respectively which can be measured at these components

This study used a LPM of the human circulation, similar to that used by Fischer et al. [16], but modelled on computer, as a physiological teaching tool (see Fig. 1) [17]. This model was selected for its simplicity; each element could be easily understood providing insight into the dynamics of the CVS, it was validated and had been used in earlier work to model heart failure severity [18, 19]. According to Shi [20] LPMs “assume a uniform distribution of the fundamental variables (pressure, flow and volume) within any particular compartment (organ, vessel or part of vessel) of the model at any instant in time” and are based upon ordinary differential mathematical equations. Other, more complex, distributed parameter models in 1-, 2- or 3-D recognise the variation of these parameters in space and use partial differential mathematical equations to describe arterial pressure and flow. Thus the different dimensional levels can be considered in the following way; a LPM can be thought of as representing a time-dependent function as a single region in space, a 1-D model as a line modelling various points along the length (x axis) with time, a 2-D model as a plane modelling various points along length (x axis) and width (y axis) with time and a 3-D model extends across all three planes modelling various points along length (x), width (y) and depth (z axis). A LPM can elegantly represent the human heart and peripheral circulation as an electrical analogue, with a capacitor (C) representing the elastic property of the large arteries (total arterial compliance) and a resistor (R) representing the frictional loss in the smaller vessels (systemic vascular resistance). The pumping chambers are represented by variable capacitors and the valves by diodes, ensuring a unidirectional flow of current representing blood flow. To model hypertension (HTN) the resistance can be increased, to model haemorrhagic shock (HS) the volume of blood in the system can be reduced and to model heart failure (HF), the contractile performance of the left ventricle can be reduced. Such models are validated and freely available from the online CellML model repository and can be run using OpenCell software (https://www.cellml.org/tools/downloads/opencell) which is, similarly, freely-available.

### The medical school

The University of Sheffield Medical School (UoS MS) has around 250 students in each of the 5 years of undergraduate study, divided into 4 phases, phase 1 “introductory clinical competency” e.g. medical sciences, phase 2 “basic clinical competency” e.g. clinical attachments, phase 3 “extended clinical competency” e.g. women and children’s health and phase 4 “advanced clinical competency” e.g. shadowing junior doctors. The UoS MS curriculum was reviewed, identifying key areas such as blood pressure control, the cardiac cycle and defining cardiac output, which the model was able to help explain. Relevant core clinical problems such as breathlessness and chest pain, were also identified to complement the basic science and also inform the interactive cases.

### Content development

**Fig. 2:**
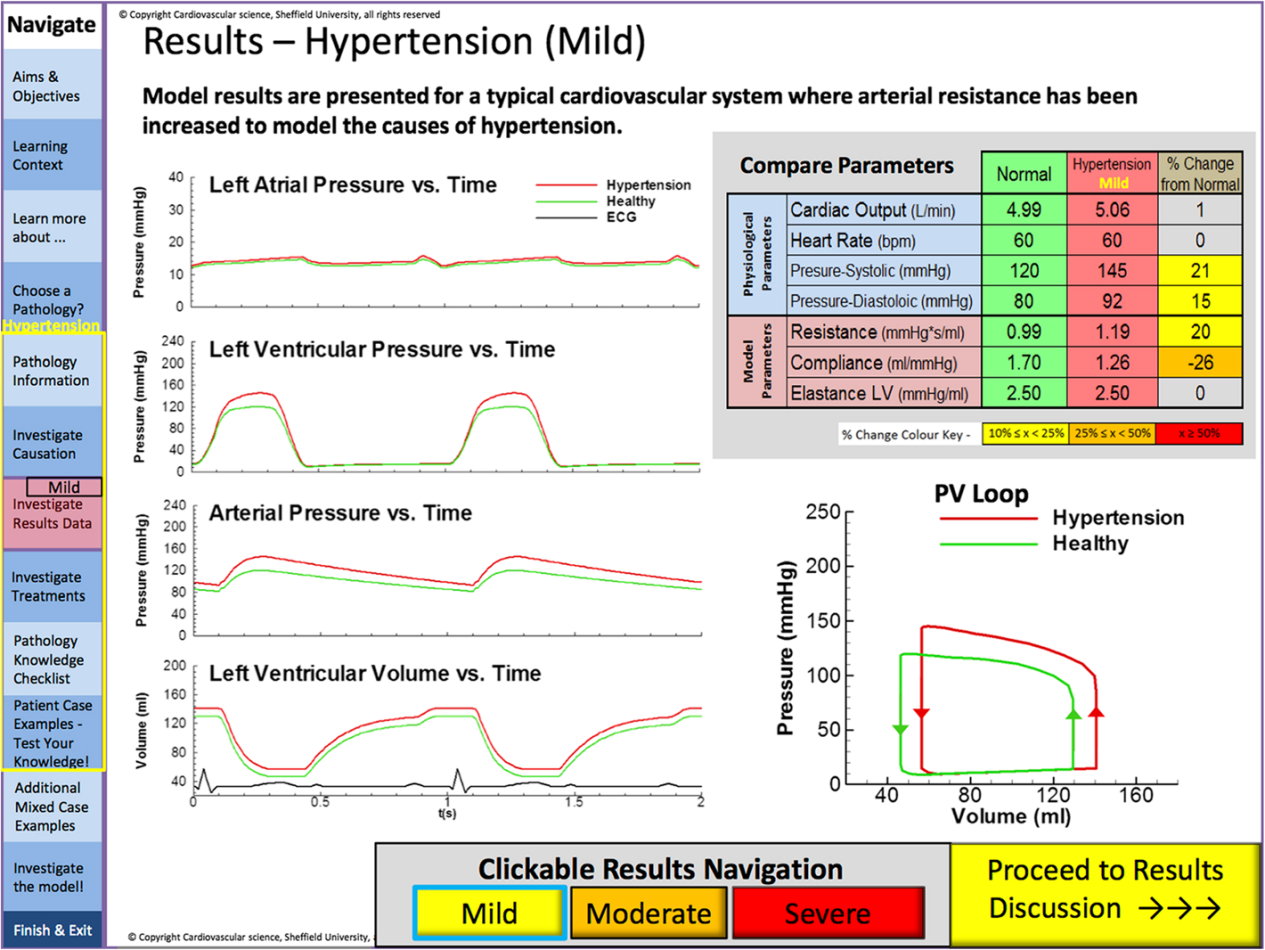
Screen shot of the environment displaying lumped parameter results in mild hypertension. The left hand side displays pressure traces in the atria, ventricle and systemic arterial system in mild hypertension (red), with comparison with a healthy adult (green) and ECG in black. The table in the top right provides a comparison of the physiological and modelled parameters in a healthy adult (green), hypertension (red) and % change from baseline (yellow). The lower right figure shows these changes as a pressure volume loop. At the bottom users can choose to view the results from models of mild, moderate or severe hypertension

The clinical topics, HF, HTN and HS were chosen as a focus of the model and to form a basis for the ELE. Up-to-date clinical guidelines on these topics were reviewed and, along with previous work, used as the basis for the models, to ensure that the outputs of the model (blood pressure (BP), stroke volume (SV) and heart rate (HR)) accurately represented different stages of each disease as described by the guidelines (see Tables 1 and 2) [19, 21–23]. This had the additional benefit of helping the students become familiar with clinical guidelines, categorisation of disease types and severity criteria. To ensure timeliness when engaging with the environment and to minimise possible difficulties in using the model, outputs were pre-computed before exposing the environment to the students (i.e. the model was not solved in real time when using the environment). The model results were created using baseline parameters (representing normal human physiology) and the input variables were then tuned (as mentioned above) to match those typically found in patients in each stage of each disease. For example mild, moderate and severe HTN were simulated by gradually increasing the systemic resistance R (see Table 1). Input variables and model outputs were displayed to the student in both tabular and graphical formats, the latter resulting in a left ventricle pressure-volume loop (see Fig. 2).

**Table 1:** The input variables used to model the various pathophysiological processes with the LPM

Inputs						
Pathological Process		LVEmax	R	C	Volume	T
		mmhg/ml	mmhg.s/min	ml/mmhg	(ml)	(s)
Normal		2.5	0.094	1.7	5700	1
Hypertension	Stage 1	2.5	1.194	1.26	5700	1
	Stage 2	2.7	1.374	1.16	5700	1
	Accelerated	2.7	1.614	1.16	5700	1
Heart Failure	Mild	2	1.094	1.7	5700	1
	Moderate	1.4	1.094	1.65	5700	0.857
	Severe	0.8	1.194	1.55	5700	0.75
Haemorrhage	Stage I	2.8	0.994	1.7	5200	0.6
	Stage II	3.2	1.034	1.7	4500	0.5
	Stage III	3	1.034	1.7	3700	0.429
	Stage IV	3	1.018	1.7	2850	0.375

*C* Total arterial compliance, *LVEmax* Maximal left ventricular elastance, *R* systemic vascular resistance, *T* duration of each heartbeat (i.e. 60/heart rate), *Volume* circulating blood volume

**Table 2:** The output parameters produced by the LPM for the various pathophysiological processes

Outputs						
Pathological Process		Guideline Derived Definition	Blood Pressure (mmHg)		Cardiac Output	Heart Rate
			Systolic	Diastolic	(l/min)	(bpm)
Normal		N/A	120	80	5	60
Hypertension	Stage 1	140–159/90–99 mmHg	140	80	5	60
	Stage 2	>160/>100 mmHg	160	90	5	60
	Accelerated	>180/>120 mmHg	180	100	5	60
Heart Failure	Mild	LVEF <50%	120	80	4.5	60
	Moderate	LVEF <40%	110	70	4	70
	Severe	LVEF <30%	100	60	3.5	80
Haemorrhage	Stage I	<15% volume loss	120	80	5	100
	Stage II	15–30% volume loss	100	70	4	120
	Stage III	30–40% volume loss	80	60	3.5	140
	Stage IV	50% volume loss	60	50	3	160

*Bpm* beats per minute, *l/min* litres per minute, *LVEF* left ventricular ejection fraction

Three virtual cases were developed for each pathology to map the model information and help the students place the physiology and model results in clinical context. Each comprised a brief history, examination findings and previous investigations where relevant. Questions were posed to the user on diagnosis, treatment, pathophysiology, severity of disease and how disease was represented within the model.

### Building the environment

The environment was built using a slide presentation platform (Microsoft Powerpoint), published as a PDF and hosted on Minerva; UoS MS’s Managed Learning Environment (https://www.sheffield.ac.uk/polopoly_fs/1.145927!/file/Minerva_eportfolio.pdf). Minerva is a web-based central resource for students and staff fulfilling many functions relating to the delivery and management of the undergraduate medical curriculum. These include information about the structure of the curriculum, timetables, course handbooks, learning materials and resources, clinical placements, student selected choice, integrated learning activities, personal portfolios, assessments and news items. It also provides access to the core curriculum database.

**Fig. 3:**
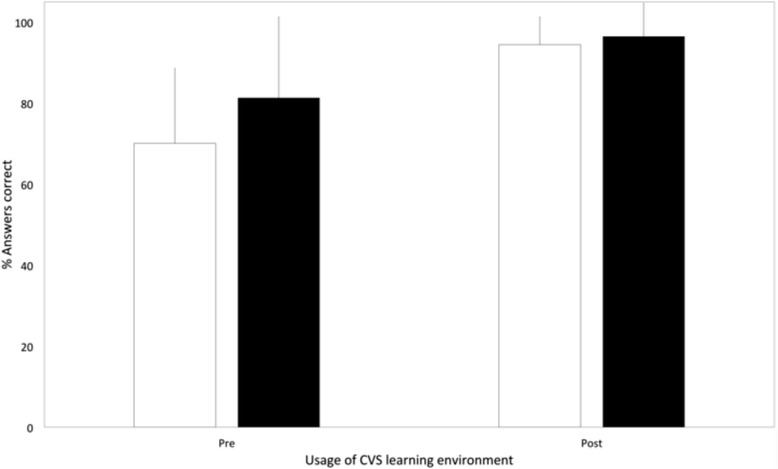
Bar chart comparing cardiovascular science knowledge before and after viewing the learning environment using Student’s T-test (black: first year medical students, white: students from all years)

**Fig. 4:**
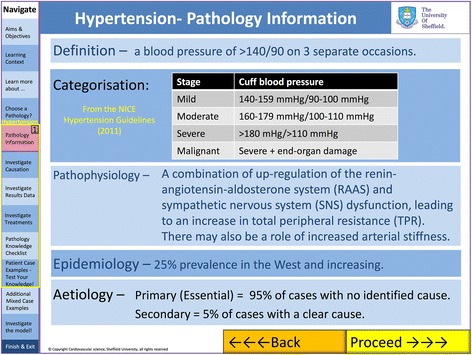
Screen shot of the electronic learning environment (ELE) demonstrating information given for each of the three clinical conditions. This includes: definition, categorisation, pathophysiology, epidemiology, aetiology At the bottom of the screen “Back” and “Proceed” buttons allow sequential navigation through the environment. The navigation tab on the left allows users to jump between areas of the ELE

**Fig. 5:**
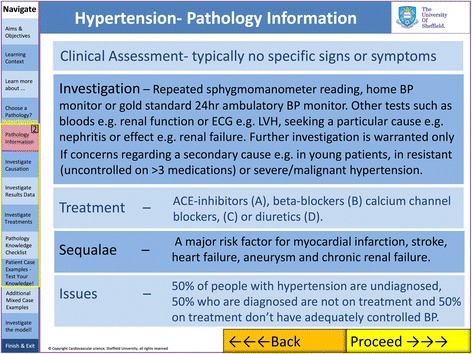
Screen shot of the electronic learning environment (ELE) demonstrating information given for each of the three clinical conditions. This includes clinical assessment, investigation, treatment and sequalae. At the bottom of the screen “Back” and “Proceed” buttons allow sequential navigation through the environment. The navigation tab on the left allows users to jump between areas of the ELE

**Fig. 6:**
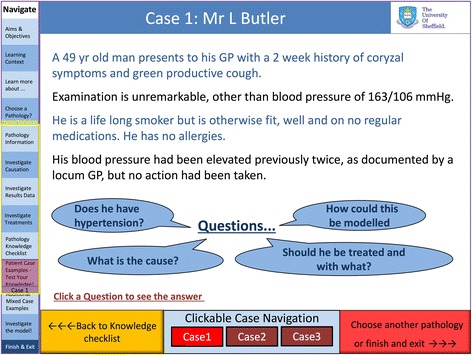
Screen shot of the electronic learning environment displaying a virtual clinical case of hypertension. The history, examination, past history and investigation outcome are provided as an introduction followed by a choice of 4 questions

The environment comprised a general introduction, modules on basic cardiovascular physiology and the three areas of pathology sub-divided into aetiology, epidemiology, pathophysiology (Fig. 3), treatments (Fig. 4), interactive model results (Fig. 2) and virtual clinical cases (Figs. 5 and 6). For each disease, questions were built into the text, both on the model parameters (for example how could one model HTN using the LPM) and then on the disease itself (for example how a patient with HF would be treated).

### Assessing and launching the environment

#### Assessment

To capture the end-users’ opinion of the environment, a questionnaire was developed, exploring the perceived relevance of the topic, the manner of delivery and the fidelity of the environment itself.

Similarly, following initial exposure to a small focus group (see below) a series of 6 questions were developed based on information contained within the environment testing knowledge of the physiology and pathophysiology of each condition. The questions were based on either a true/false or extended-matching question (EMQ) format familiar to the students, as used at UoS MS (see Additional file 1).

#### The learning environment exposed

The exposure of the ELE involved three distinct elements.

Phase one, in November 2012, a small self-selected focus group of 9 students (from all years) were recruited in response to an advert on Minerva and given a 90-min interactive tutorial, following which they were supported by the authors in use of the environment. The feedback from this session was used to revise the environment.

Phase two, the environment was demonstrated to 28 first year medical students following a routine lecture on the cardiovascular system (in December 2012), and the environment was then released to this group in January 2013, for a period of 2 weeks, after they had finished the cardiovascular taught module and before starting their first clinical placements. Feedback was gained on the environment in terms of content, delivery, style and presentation with a free text option (see Additional file 2) and the students also tested their knowledge of the CVS, based on information contained within the environment, both before and after use (see Additional file 1). This cohort was independent of the focus group (many of whom were close to graduation). Use of the environment was optional and other than the demonstration in the lecture, no other assistance was given.

Phase three, Finally, the environment was made available to the entire UoS MS. Over seventy students from all years viewed the environment, for a period of 2 weeks, gave feedback and were assessed on their learning.

### Statistics

Performance on the questions in Additional file 1, before and after exposure to the ELE was compared with statistical analysis using SPSS statistics, Version 21 (IBM, USA), using a two-tailed t-test with a confidence interval set at 95%.

## Results

**Fig. 7:**
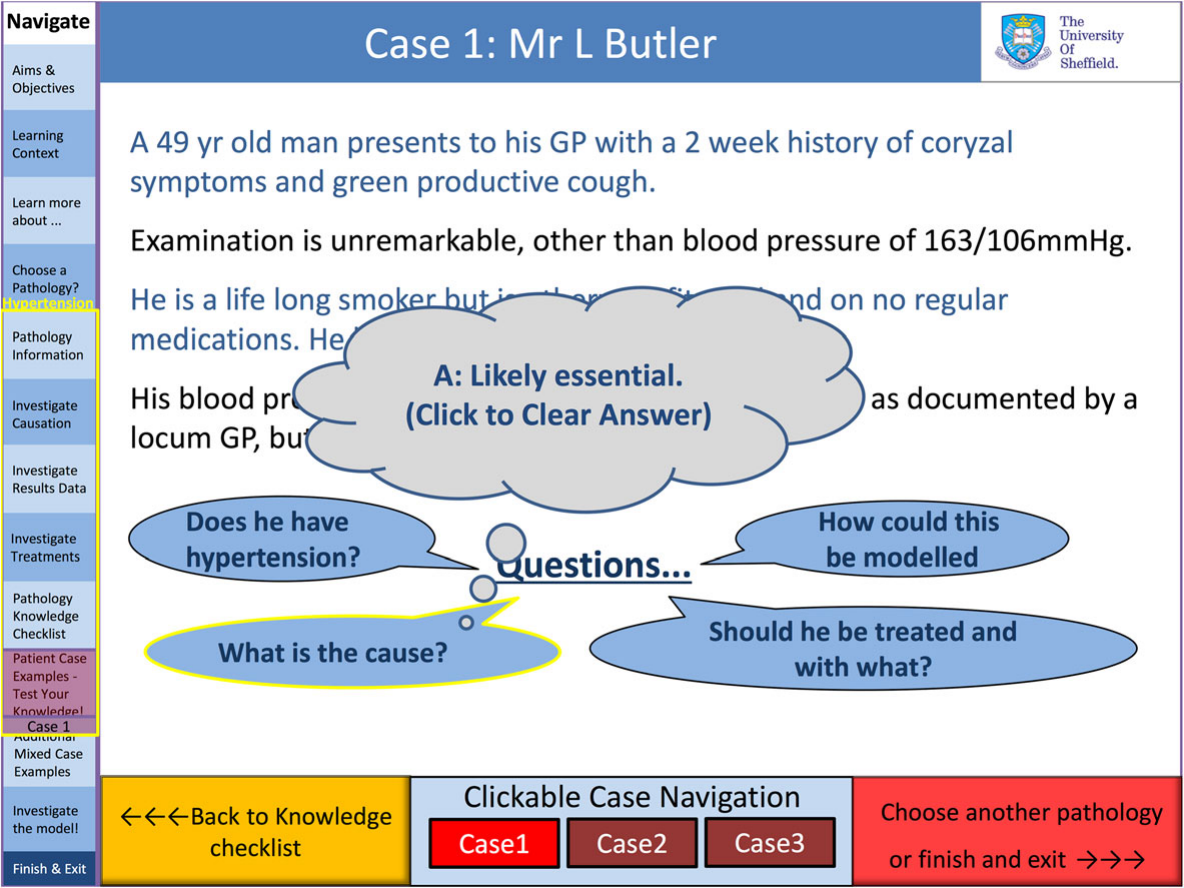
Screen shot of the electronic learning environment displaying a virtual clinical case of hypertension. The answer to a question appears (grey bubble) when the user clicks on this item

The questionnaire (see Additional file 2) provided quantitative data on the utility of the ELE whereas qualitative data was acquired (see Table 3) from free text boxes. Quantitative assessment of students’ learning was based on 6 questions covering the content of the environment. These questions were directly derived from the cardiovascular medicine component of the UoS MS undergraduate curriculum. There was a statistically significant increase in their performance after exposure to the environment, with a 33% and 22% increase in the number of questions answered correctly, *p* < 0.0001 and *p* < 0.001 respectively (see Fig. 7).

**Table 3:** The demographic data of the 3 cohorts of students and their responses to the questionnaire about the e-learning environment

Group	Focus Group (*N* = 9)		First Year (*N* = 28)		All Medical School (*N* = 76)	
Age	21–23	44%	18–20	64%	18–20	56%
Cardiology^a^	18%		36%		32%	
Physical^b^sciences	22%		36%		29%	
Question No.^c^	% Agree/ Strongly Agree	Mean Likert score	% Agree/ Strongly Agree	Mean Likert score	% Agree/ Strongly Agree	Mean Likert score
4	88	4.2	93	4.1	88	3.9
5	100	4.4	93	4.4	94	4.3
6	100	4.1	93	4.1	88	4.1
7	88	4.1	78	3.9	77	3.8
8	66	3.6	78	3.8	71	3.7
9	66	3.6	85	4.1	88	4.1
10	88	3.7	93	4.4	94	4.4
11	77	4.0	78	3.9	82	3.9
12	88	4.1	93	4.3	94	4.2
13	100	4.2	85	4.1	85	4.0
14	100	4.2	100	4.5	91	4.2
15	88	4.2	100	4.4	94	4.1

^a^Interested in a career in cardiology or cardiovascular sciences

^b^Did you study the physical sciences and

^c^see questionnaire for a full list of questions

During phase one comments from the focus group centred on minor (typographical) errors, a requirement for more information about the pressure-volume (PV) loops used to display the modelled results (many students were unfamiliar with these), a request for an increased number of clinical cases as these were well-received and, finally, a more interactive approach to Q&A scenarios making the answer to each question available via a single click (this version showed the answers to all five simultaneously on a separate page).

The students reported that they found navigation intuitive, that it successfully communicated the importance of the physical principles of the cardiovascular system and was useful for demonstrating the effects of common cardiovascular pathologies. Suggestions included making links between pages more obvious, using colour-coding to indicate disease severity and using tabs for different pathologies. These suggestions were adopted in order to improve the next iteration of the ELE.

Free text comments included – *“Excellent interactive tool”*, *“Nice the way it integrates both physiology and pathologies of the heart”* and *“CRT = means capillary refill time but then also cardiac resynchronisation therapy, this is confusing”*.

Table 3 displays the questionnaire results.

During phase two first year medical students were given access to the environment for a period of 2 weeks. They engaged well with the environment, requiring minimal guidance; none of the authors were contacted during this period for advice on how to use the environment. As the students enjoyed the clinical cases, and commented they would like more, an additional ten were added for phase 3 evaluation. The students reported that too many abbreviations were used, many of which they had not encountered before. These were explained in full or removed from the phase 3 version.

Free text comments include *“Helped me revise the physiology of heart contraction effectively”*, *“Realistic interpretation of medicine when we finally graduate”* and *“Too much information on each page”*.

During phase three the UoS MS as a whole was given access to the environment, via Minerva, for a period of 2 weeks.

Comments included *“Similar resources for respiratory and neurology would be great!*” , *“An excellent and extremely useful resource, thank you!”* and *“…could do with being less busy, rigid and dark.”*

## Discussion

This is the first study to demonstrate that a LPM can be embedded in a cardiovascular ELE, positively received by medical students and exposure to which appears to improve knowledge of specific areas of cardiovascular pathology, namely hypertension, heart failure and haemorrhage.

Other simulations exist for medical students studying cardiovascular science and cardiology, for example how to examine patients and reading of ECG’s, but as noted by Owen & Wong most simulations are used as an introduction, not a tool for repetitive practice [24–28]. Such tools allow students to increase their confidence and experience in particular skills, like diagnosing a cardiac murmur or recognising a particular cardiac arrhythmia. Our work was aimed specifically at increasing medical students knowledge of cardiovascular physiology and pathology using an ELE based upon a simple LPM and then applying this to the diagnosis and management of relatively low fidelity simulated patients e.g. text descriptions only.

Many studies, including this one, lack a direct comparison to pre-existing practice. For example student performance may improve following exposure to a simulation, but they are seldom randomised to one methodology or another. Only two studies were identified as having done this. Firstly, Sverdup et al. (2010) randomised students to traditional bedside teaching versus computer simulated heart sounds, and indeed found no difference in performance between third year medical students at Oslo University Medical School [29]. Kern et al. (2011) used students from previous years as controls and provided simulated patients in addition to, not instead of, traditional training and so it is difficult to tease apart the influence of the simulations, even though performance was improved [24]. Indeed, as demonstrated by Lavaranos, simply introducing an intensive traditional bed-side cardiology course may be sufficient to improve learning, rather than hi-fidelity simulations [30]. Further questions arise related to ELE effectiveness such as how much is retained in the short, medium and long term and how performance and confidence in clinical practice following graduation is altered? This is highlighted by the study by Vulkanovic-Criley [31], who demonstrated that even following the completion of clinical training in cardiology, qualified doctors may perform no better than their medical student counterparts, despite the exposure to an ELE comprising of case vignettes of patients with cardiovascular pathology. Many studies, including this one, were conducted at a single academic institution, with only one study using multiple centres; this comprehensive study was undertaken by Vulkanovic-Criley et al. [31], who tested over 860 participants, including 318 medical students, across multiple centres, on cardiac physiology, auditory skills, visual skills and integration of both auditory and visual skills using computer graphic animations and assessments of virtual cases. However, this was not designed to improve knowledge of skills e.g. it was not an ELE, but merely test them. Intriguingly, they found that whilst cardiac examination skills improved during medical school (from first to third year), they did not improve or differ significantly following the third year of medical school, following qualification or indeed across any group of doctors, regardless of seniority or speciality. Only heart specialists, cardiologists, had significantly better cardiac examination skills compared to the rest of the groups.

Petrusa et al. [32] document the introduction of a 4 year multimedia curriculum in cardiology, into 4 medical schools, at significant cost, comprising of “ten interactive, patient-centered, case-based modules focused on the history, physical examination, laboratory data, diagnosis, and treatment” and whilst 80% of the students rated the system highly, a similar number to this study, there was no comparison with previous teaching delivery and so there is an implicit assumption that this is better, without any measurement, either of students performance or indeed how highly the old system was rated.

The most similar previous work to this study is Dassen et al. [15], who adopted a similar approach based on the ‘CircAdapt’ model, for first year medical students in Maastrict, but are yet to publish outcome data on how students respond to, or learn from, such a model. Such information is critical to this type of model, or environment development, as without user-feedback or evidence of improvement following use, the models cannot be regarded as valid tools for teaching physiology. Furthermore, whilst the Dassen method involves a more complex but stand-alone model, it also requires background information to be provided during face-to-face teaching. The ELE described here has the advantage that it does not require real-time teacher-student supervision and was tested by the whole medical school. It also includes an integrated Q&A component to test the students understanding of the model, CV physiology and pathophysiology. The simulated clinical cases play an important role in bringing this information together. On the other hand, the Dassen method allows the model to be run in real-time; if sensible physiological boundaries were set this could also be done for our ELE, enabling students to choose their own combinations of vascular resistance, vascular compliance and cardiac performance.

A delicate balance had to be achieved between adding detail to the model (with the risk of bewildering those students with little underpinning knowledge of physics) or embedding the model so deeply within the environment that its purpose was lost (see Figs. 5, 6 and 7). It is interesting to consider; ‘would the ELE be as useful if the model was removed?’ The interactive clinical cases and the outputs of their respective models helped maintain a sense of realism and a clinical relevance and enabled the students to test their knowledge and understanding in a safe environment. Consequently we believe that a good balance was reached in this respect and consider that the model provided a platform on which to build the environment, allowing insight into the processes underlying the pathology without which the experience would have been poorer.

The process of development was necessarily an iterative one, drawing on previous experience of such environments within the department. The main lesson learned from previous environments, was that the target audience and purpose of environment must be identified a *priori*, otherwise a seemingly excellent environment could be developed that was subsequently found to be either too complex for undergraduate medical students or contained too much basic science for cardiac specialists.

At each stage feedback from user-directed improvements ensured that the environment was relevant, appropriate and interesting. Feedback was generally positive, with criticisms limited to presentation, rather than the detailed content or concept as a whole. Similarly, the environment was endorsed by both cardiology academics and medical educationalists at the UoS Medical School, who deliver cardiovascular science lectures and design the curriculum, respectively.

The collaborative effort including an e-learning technologist, cardiovascular modellers and a cardiologist with an interest in education, meant that the three key areas of the developmental process, identification of what could be modelled, what was relevant to model and the method of delivery of model output were discussed contemporaneously rather than retrospectively. This led to a streamlined development process.

As presented in the results, there was a significant improvement in the number of questions answered correctly after exposure to the ELE, suggesting that the students benefited from the experience. Furthermore, an improvement in baseline results (before exposure) can be seen, between iterations of the model. Notably, the 2nd iteration was available to only first year medical students but the 3rd iteration was accessible to all year groups; it would be expected that their level of clinical knowledge, and therefore their performance, would be much higher.

From a technical perspective it became challenging to maintain a structured design of the ELE in its final iteration, which contained over 200 pages. However the user was not required to interact with each page sequentially and no criticisms were recorded in this regard. Choosing how and what to display from the range of possible outputs of the model required a fine balance. Although the students had limited exposure to the concept of PV loops, they had had some exposure to the significance of measures such as SV, BP and HR and the PV loop is an excellent way of graphically demonstrating relationships between cardiovascular parameters. Abbreviations commonly understood by the focus group, proved not to be immediately transparent to first year medical students. As a result, nearly all of the abbreviations were removed from the second iteration of the software with a key provided to aid clarity. Ideally, students would be granted unlimited access to the environment, both in terms of time and usage, but due to time constraints the students were restricted to an access period of 2 weeks after which the link was removed. In addition the software was not downloadable. It is possible that this may have limited uptake and interaction.

The model chosen was a simple LPM, but there are many more complex models of the heart and peripheral circulation available; these include 2D, 3D as well as other more complex 0D representations. Whilst the inclusion of a more complex 3D model might yield more complex outputs there is a danger that the software would become slow and unwieldy and ultimately, 3D solutions may not add anything over and above the simple model.

In the current environment the results of the model were pre-computed. The model was run with a range of typical inputs and the user had access to a pre-specified set of results. In the future, it would be possible to allow the students to run the model in real time, offering infinite possibilities but boundaries would have to be set to prevent results that are physiologically improbable or impossible.

The assessment questions were provided in a format familiar to medical students through the routine MBChB audit of teaching quality. As seen in Table 3, it is notable that whilst all users were of a similar age, less than 30% either had a predilection for cardiology or had studied physical sciences at either pre-university or undergraduate level, demonstrating just how accessible the ELE was without necessarily needing prior knowledge or indeed a special interest.

### Future work

The simulated patients could be further developed, allowing history-taking, examination findings, results from investigations and treatment options, all interacting with the model [33–35]. Other opportunities involve tailoring the environment to the British Cardiology Society (BCS) curriculum for cardiologists in training or collaborations with colleagues from other specialties and scientific disciplines for the development of similar environments, in respiratory medicine for example. This study focused on cardiology which is perceived as one of the most interesting specialities. The general approach has potential to be extended to focus on specialties which students and doctors struggle to engage with, neurology for example, but evidence suggests performance across these subjects is actually similar [36, 37].

One could foresee such an environment being downloadable to a smart phone or tablet device, which may increase engagement and perhaps allow users to edit the environment and upload improved and updated versions. Customisation could even enable them to create their own simulated patients with questions to test their peers.

Comparison of performance before and after using the model and comparing this with performance before and after lectures on the same subject would be a useful comparison of teaching methods.

The effects of therapies on cardiovascular performance e.g. administration of IV fluids, b-blockers, or use of biventricular pacemakers could also be included. Finally, one could envisage the development of a series of informal teaching aids with students downloading an environment, carrying out beta-testing of the software, and then uploading it for others to use.

Clinicians and academics also acknowledge the utility and future possibilities of such environments and as such, there is enthusiasm locally to integrate these types of applications into the undergraduate curriculum.

### Limitations

The questionnaire used to assess the ELE was not validated, but was based upon the UoS MS curriculum, which in turn was incorporated into the material on the cardiovascular system for the ELE used in this study and so the results must be viewed in that regard. Only 15 students provided free text responses and so the generalisability of this form of feedback is limited. No attempt was made to investigate potential impact on examination performance or to investigate if exposure to the model encouraged more medical students to choose a period of research in the cardiovascular sciences. There were also many questions embedded in the environment for which responses were not recorded and so it would have been useful to record performance during usage to see whether knowledge improved during progression through the environment. There are some possible confounding variables, for example it was not feasible to restrict access to the ELE during testing and so it is possible some students treated the assessment like an open book, the testing reflecting their ability to access information in the ELE not whether they were able to recall and retain the same information from the ELE. Also, whilst knowledge of the cardiovascular system appeared to improve, ultimately it is understanding of the cardiovascular system that educators seek to enhance, as noted by Dullo and Chaudhary [38], especially as some students may understand less than they know. Finally, the sample size was small, not randomised, conducted at a single institution, not powered to detect changes in knowledge following exposure to the ELE and was also not directly compared to other learning modalities, or indeed, the students existing one.

## Conclusions

LPMs are valid components of CVS teaching tools when used in conjunction with a novel and interactive electronic learning environment (ELE). ELEs enable students to interact with, and manipulate, physiological processes that occur in response to disease. Rather than simply reading how the physiological and pathophysiological processes happen, users can study this interactively. In this study, we discovered that students found using this LPM embedded in this particular ELE an interesting and positive experience. Even from a solid foundation of traditional teaching input, considerable improvements were made in students’ knowledge during their assessment. This work combines some exciting and developing fields in medical education, namely modelling, e-learning and simulated patients and illustrates the benefits that can be achieved with engagement of educationalists, clinicians and modellers.

## Additional files

Additional file 1: Questions to test the users knowledge of the cardiovascular system based on information contained in the e-learning environment, assessed before and after exposure. (DOCX 13 kb)

Additional file 2: Learning environment questionnaire to explore the perceived relevance of the topic, the manner of delivery and the fidelity of the environment itself. (DOCX 14 kb)

## Abbreviations

BMJ: British Medical Journal
BP: Blood pressure
CV: Cardiovascular
CVS: Cardiovascular system
ECG: Electrocardiogram
ELE: e-learning environment
EMQ: Extended matching questions
ESC: European Cardiology Society
HF: Heart failure
HR: Heart rate
HS: Haemorrhagic shock
HTN: Hypertension
LPM: Lumped parameter model
MS: Medical School
Q&A: Question and answer
SV: Stroke volume
UoS: University of Sheffield
