# Supplementary material for: Computer model for the cardiovascular system: development of an e-learning tool for teaching of medical students
Source: BMC Med Educ. 2017 Nov 21;17:220. doi: 10.1186/s12909-017-1058-1 (PMC5697416; doi:10.1186/s12909-017-1058-1)
Supplement: Supplementary file 1 — Questions to test the users knowledge of the cardiovascular system based on information contained in the e-learning environment, assessed before and after exposure. (DOCX 13 kb) [file 12909_2017_1058_MOESM1_ESM.docx]

Additional file 1: Questions to test the users knowledge of the cardiovascular system based on information contained in the learning environment, assessed before and after exposure.

1. Hypertension is due to up-regulation of the renin-angiotensin-aldosterone system (RAAS) and sympathetic nervous system (SNS) dysfunction, leading to a decrease in total peripheral resistance (TPR).

True False

2. Haemorrhagic shock is a sudden loss of blood from the cardiovascular system. Resulting in a triad of -

A: Hypotension, tachycardia and increased organ perfusion.

B; Hypertension, bradycardia and increased organ perfusion.

C; Hypotension, tachycardia and decreased organ perfusion.

3. Heart failure is a diagnosis based on the presence of impairment of left ventricular function alone.

True False

4. The majority of cases of hypertension have a clear and treatable cause.

True False

5. If you can palpate both the carotid and the femoral pulse, but not the radial, what is the approximate systolic blood pressure.

A: 60-70 mmHg

B: 70-80mmHg

C: 80-90 mmHg

6. A patient presents with symptoms of breathlessness on minimal exertion and an echocardiogram which demonstrates a left ventricular ejection fraction of 35%. What is the severity of their symptoms according to the NYHA classification and also the severity of their left ventricular systolic dysfunction

A: NYHA class IV and Moderate LVSD.

B: NYHA class III and Moderate LVSD.

C: NYHA class III and Severe LVSD.
