# Supplementary material for: Computer model for the cardiovascular system: development of an e-learning tool for teaching of medical students
Source: BMC Med Educ. 2017 Nov 21;17:220. doi: 10.1186/s12909-017-1058-1 (PMC5697416; doi:10.1186/s12909-017-1058-1)
Supplement: Supplementary file 2 — Learning environment questionnaire to explore the perceived relevance of the topic, the manner of delivery and the fidelity of the environment itself. (DOCX 14 kb) [file 12909_2017_1058_MOESM2_ESM.docx]

Additional file 2: E-Learning Environment Questionnaire to explore the perceived relevance of the topic, the manner of delivery and the fidelity of the environment itself.

Background Information:

1) Your Age Category:

18-20 21-23 24-27 27-30 30+

2) Do you have an interest in cardiovascular science or becoming a cardiologist?

Yes No Unsure at this stage

3) Did you study physical sciences e.g. engineering or physics, at A-level or above?

Yes No

Clinical Knowledge:

1) This resource successfully communicates the importance of physical principals to the cardiovascular system.

Strongly agree Agree Neutral Disagree Strongly Disagree

2) This resource is valuable in demonstrating the causes of common cardiovascular pathologies.

Strongly agree Agree Neutral Disagree Strongly Disagree

3) This resource is useful for demonstrating the range and appropriateness of treatments for common cardiovascular pathologies.

Strongly agree Agree Neutral Disagree Strongly Disagree

4) The clinical descriptions in the resource are exactly relevant for my level of clinical education.

Strongly agree Agree Neutral Disagree Strongly Disagree

5) The amount of technical detail in the resource is exactly relevant to my level of clinical education.

Strongly agree Agree Neutral Disagree Strongly Disagree

6) The Introduction and background information included in the resource is sufficient to enable my understanding of the resource.

Strongly agree Agree Neutral Disagree Strongly Disagree

7) The interactive multiple-choice questions in the resource were useful to my understanding of the subject.

Strongly agree Agree Neutral Disagree Strongly Disagree

8) The pathology specific model results sets were useful to my understanding of the subject.

Strongly agree Agree Neutral Disagree Strongly Disagree

9) The pathology specific sets of mini-cases were useful to my understanding of the subject.

Strongly agree Agree Neutral Disagree Strongly Disagree

10) The amount of information presented per page was reasonable.

Strongly agree Agree Neutral Disagree Strongly Disagree

11) It was obvious how to navigation through this resource.

Strongly agree Agree Neutral Disagree Strongly Disagree

12) The sequence in which the information was organised for the learner was natural and well thought out.

Strongly agree Agree Neutral Disagree Strongly Disagree

13) The Virtual Physiological Human Initiative provided the funding and model data for this resource- had you heard of the Virtual Physiological Human (VPH) prior to using this resource?

Yes No

14) Please use this comment box to suggest any improvements you can think of…. (free text box)
